# Treatment effects in orthopaedic trials are underestimated by applying patient‐level PRO thresholds for meaningful differences at the group level

**DOI:** 10.1002/ksa.12805

**Published:** 2025-09-08

**Authors:** David F. Hamilton, Karlmeinrad Giesinger, Johannes M. Giesinger

**Affiliations:** ^1^ Research Centre for Health Glasgow Caledonian University Glasgow UK; ^2^ Department of Orthopaedic Surgery Kantonsspital St. Gallen St. Gallen Switzerland; ^3^ Medical University of Innsbruck, University Hospital of Psychiatry II Innsbruck Austria

**Keywords:** minimally important difference, orthopaedic trials, patient‐level PRO thresholds, group‐level PRO thresholds, patient‐reported outcomes

## Abstract

Orthopaedic trials frequently rely on patient‐reported outcomes (PROs) to measure primary end points. Thresholds for clinically meaningful score differences are then used to interpret PRO scores and support result interpretation. At the patient level, thresholds are used to determine if an individual patient has experienced a clinically meaningful improvement or deterioration, which evaluates whether or not they are a treatment responder. At the group level, thresholds are applied to interpret mean score differences between groups (e.g., trial arms) or between time points and determine if a treatment effect is meaningful. While patient‐level thresholds are frequently available for commonly used PROs, interpretation of between‐group‐level differences is far less established. In the absence of well‐defined group‐level difference thresholds for PRO scores, patient‐level thresholds are frequently used to interpret the difference between groups, such as trial arms. However, meaningful difference thresholds at the patient level are typically larger than relevant differences at the group level. As such, this leads to an underestimation of treatment effects reported in orthopaedic trials.

**Abbreviations**
CMD: clinically meaningful difference
PRO: patient‐reported outcome
PROM: patient‐reported outcome measure

## INTRODUCTION

Patient‐reported outcome measures (PROMs) are routinely used in orthopaedics to quantify clinical outcomes; as the variables that we are typically interested in, such as pain or treatment satisfaction, are only accessible through these tools. Reflecting this, PROMs are frequently the preferred primary outcome measures for orthopaedic clinical trials. The primary trial end point is the parameter that the investigators consider most important in answering the study question. As such, the primary endpoint is all‐important, essentially determining whether the trial intervention was effective or not [15, 27]. To make this critical ‘success’ decision using a PRO end point, we need to know what number of score points on a questionnaire scale constitutes a meaningful treatment effect, that is, a ‘meaningful difference’ between trial arms over time.

Clinically meaningful differences (CMDs) are a critical concept when interpreting the relevance of PRO results [13, 19]. These thresholds are classically defined as ‘the smallest difference in score in the outcome of interest that informed patients, or informed proxies, perceive as important, either beneficial or harmful, and which would lead the patient or clinician to consider a change in the management’ [24], or put more simply, as ‘any amount of change that is considered meaningful and worthwhile by the patient’ [9]. There are multiple terms used to describe various PRO thresholds interpreting clinical meaningfulness, for example, minimal clinically important difference, minimal important difference or clinically meaningful change. Confusingly, the technical definitions applied to these terms differ substantially throughout the literature [14, 16]. However, they mostly refer to the same basic concept. For simplicity, in this article, we use the generic term ‘clinically meaningful difference’ (CMD) and discuss these concepts for score change in individual patients and score differences between groups (or treatment arms in clinical trials).

CMD thresholds have been reported for most of the established orthopaedic and general health PROMs, and these values are regularly used to power and interpret clinical trials. There are, however, poorly understood limitations with the CMDs that are routinely employed. A critically important, but frequently ignored issue is the commonly seen application of patient‐level CMD thresholds (designed to interpret the score change that matters to the individual patient) at the group level; to determine whether an intervention has achieved a relevant difference from the comparator arm in a trial [1, 21, 23]. This may lead to incorrect interpretation of trial results through an underestimation and undervaluation of the clinical relevance of the outcome score difference found between trial arms.

## INDIVIDUAL PATIENT CHANGE VS. THE AVERAGE CHANGE OF A GROUP OF PATIENTS

Patient‐level and group‐level CMD thresholds are not the same thing. Patient‐level CMD thresholds interpret data relating to the score change achieved by an individual patient (e.g., patient X has improved by 10 points on a pain scale between the preoperative and 6‐month follow‐up assessment). Group‐level CMD thresholds interpret the average difference between groups (e.g., differences between the mean change of patient groups from preoperative to 6‐month post‐operative follow‐up). Describing what an individual patient considers to be a meaningful change helps to identify threshold values at which patients experience a treatment benefit. This allows categorisation of patients as treatment responders or not. However, these patient‐level CMD thresholds should not be mistaken for the mean score values at which relevant group‐level differences have occurred. A true group‐level threshold is neither part of the end point definition, nor of the statistical analysis, but is a means of interpretation applied after the analysis of the trial data to deduce clinical meaning regarding relevance from the trial results.

At the patient level, CMD thresholds are established by using an external anchor that reflects how much change has occurred in the patient's symptoms and whether they feel better or not. Here, patients are assessed with a PROM at two time points, and at the later time point, an additional ‘global‐change’ question asks whether the patient has perceived a change in status relative to the first time point (e.g., that they are doing: much worse, a little worse, the same, a little better and much better). This allows calculating the magnitude of change in points of the PROM that reflects when the average patient perceives a change to have happened (i.e., reports being at least a little better). Through statistical methods (typically receiver‐operating characteristic curves or logistic regression modelling), a CMD threshold can subsequently be derived. Alternatively, distribution‐based thresholds (often taken as 0.5 standard deviations of the PROM population mean score) may offer a useful estimation when external anchors are not available to calculate robust values [22, 28, 29].

While such methodology is well‐established for calculating patient‐level CMD thresholds, there is little methodological agreement as to how to establish group‐level CMD thresholds that can guide the interpretation of group‐level differences. The use of group‐level anchors has been recommended, using expert ratings as to the magnitude of mean differences between known group comparators [6, 7]. However, this may be challenging to operationalise and has not been achieved with orthopaedic‐relevant PRO scores yet.

In the absence of well‐defined group‐level anchors for orthopaedic PROMs, the German Health Technology Assessment body, the Institut für Qualität und Wirtschaftlichkeit im Gesundheitswesen (IQWiG, Institute for Quality and Efficiency in Healthcare) [12] currently advises taking a distribution estimate of 0.2 standard deviations as being reflective of a relevant group‐level difference. This approach is similar to the classification of effect sizes, such as Cohen's *d* [8]. Interestingly, this approach has not, as yet, been adopted in orthopaedic clinical research. Where investigators have tried to establish group‐level thresholds in orthopaedic scores, there are issues when translating these to the trial setting. The most common approach is by estimation of the mean differences between different categories of an external anchor in a cohort of patients [2, 5]. For example, Beard et al. [2] reported the group‐level CMD for the Oxford Scores to be 5 points, based on the difference in the mean change of the OHS and OKS in patients who identified themselves to be ‘a little better’ and ‘about the same’ on a patient‐reported global transition item (anchor question). While this approach clearly refers to group‐level data, the value obtained informs as to the mean difference between a group in which all patients report no change and another group in which all patients report ‘a little change’. This does not align particularly well with a clinical trial, where the experimental arms are expected to comprise a range of patients, who may individually experience no change, a little change, or larger magnitudes of change. The statistical approach to calculating threshold values that accompanies this methodology further highlights the issue. Unlike logistic regression models, mean differences are not intended to provide classification thresholds [25] but rather to inform differences between the centres of distributions. These CMD values however are applied with the expectation that a meaningful difference exceeds the threshold, while differences below it are seen as trivial.

As currently there is no methodological consensus as to technically calculating group‐level CMD thresholds for PROMs, it is reasonable to assume that the magnitude of PRO score points constituting patient‐level and group‐level CMD thresholds will inherently be very different. Importantly, a meaningful patient‐level difference would typically be of a larger magnitude of PROM score points than the value required for a relevant group‐level difference, because the mean change in a PRO at the group level is the average change of a group of patients who do and do not achieve a CMD.

## ASSESSING BETWEEN‐GROUP DIFFERENCES IN CLINICAL TRIALS

A classic two‐arm randomised controlled trial evaluates the outcomes of an intervention group versus those of a control group. It is reasonable to expect some variation in treatment response among the trial participants, and that a certain proportion will benefit from the intervention/treatment (i.e., be treatment responders) while some will not. Further, assuming the trial intervention affects the outcome investigated, it is also reasonable to expect that the different arms of the trial will result in different numbers of treatment responders. If the intervention is beneficial, a larger number of treatment responders will be seen in the intervention group, and this will result in a larger group‐mean change score in favour of that trial arm compared to the control arm.

However, it is critically important to highlight that the group‐level mean change in a trial arm always reflects both the responders and the non‐responders. Because the group‐level mean reflects the proportion of both responders and non‐responders in the group, the trial intervention effect would need to be very large for a between‐group mean difference to exceed the patient‐level CMD threshold of a PROM. In effect, at group‐level, the scores of the non‐responder proportion in the trial arm ‘dilute’ the change score reported by the responder proportion. It follows then, that observing a trial between‐group difference that is lower than the accepted patient‐level CMD threshold for a PROM does not necessarily mean that a group‐level (or societal‐level) relevant meaningful change has not occurred through the study intervention/treatment.

To make these arguments more tangible, it may be more intuitive to consider other types of outcomes, like blood pressure or range of motion, to illustrate this concept. An average reduction in systolic blood pressure of 4 mmHg as a result of a new drug, compared to standard treatment, might suggest a substantial advancement in the treatment of resistant hypertension [30], and the drug would likely be accepted as a useful intervention and prescribed. However, for an individual patient, a reduction of 4 mmHg would be considered irrelevant. At the patient level, a very much larger reduction of 20 mmHg [4] is considered a meaningful CMD threshold for the evaluation of a successful treatment. This illustrates how an intervention can produce an effect of a magnitude that is meaningful at the population level but that may not be perceived as clinically significant by an individual. It is well documented that the prescription of anti‐hypertensive medication at an early stage is generally considered to have been a great success in reducing blood pressure and lowering cardiovascular risk. Recent evaluation of data from the United Kingdom and the United States evaluating the change in blood pressure between 1992 and 2019 as a result of antihypertensive medication offers a pooled estimate of around 9 mmHg reduction in systolic and 6 mmHg in diastolic blood pressure [18]; a group‐level improvement enhancing population health, probably contributing to better overall healthcare outcomes and costs—but likely not relevant to the average individual.

Similarly, as a more orthopaedic example regarding range of motion, a mean difference of 5° between two study arms in a clinical trial as a result of an intervention may be considered to represent a relevant difference. If we can confidently say that a new implant or rehabilitation regime allows patients' knees to bend 5° more, on average, than our existing standard‐of‐care, at a societal level, we will likely accept that as being generally beneficial and introduce that intervention/regime (assuming that it is cost‐effective). However, a range‐of‐motion increase of 5° may be felt to be of little importance during activities of daily living, and may even not be perceptible, to any given patient [26].

## ASSESSING WHETHER AN INTERVENTION MAKES A GROUP‐LEVEL DIFFERENCE

The incremental evolution of orthopaedic technology and surgical technique typically offers subtle benefits over previous generations. If we primarily evaluate the group‐level success using a PROM score change value that is ‘meaningful’ to the average individual, we necessitate that the new intervention in question produces a very substantial treatment effect to be classed as successful and relevant. As a result, we may be missing the wider relevance of beneficial technologies, techniques, or treatment regimes. Determining the clinical relevance of group‐level differences should not just be based on the magnitude of change that individual patients consider to be important, but on the broader context of the health problem and its available treatments, along with a careful evaluation of risk versus benefit by all relevant stakeholders [11].

Various international guidelines instruct as to the use of PRO data in clinical trials [3, 6, 10, 17, 20]. There is increasing awareness as to the distinction between, and implications of, patient‐level and group‐level CMD thresholds in terms of statistical analysis and interpretation of results [6, 20]. Ultimately, we need robust group‐level CMDs for orthopaedic PROMs to better interpret the results of comparative clinical studies. While group‐level anchors are methodologically challenging to establish, it may, for the time being, be sensible to directly interpret effect sizes of analyses of mean changes and differences in responder proportions rather than confuse patient‐level and group‐level CMD thresholds to interpret different outcomes by treatment group.

Using a PROM that assesses pain or function as the primary outcome in an orthopaedic trial, or indeed any clinical study, is a sensible choice in terms of the outcomes we are typically interested in. However, we must recognise the danger of assessing between‐group differences using inappropriate CMD thresholds, which may lead to an underappreciation of relevant differences between trial arms or in group change over time, and, consequently, to an underestimation of a clinically relevant effect having occurred. Future research should focus on developing robust, group‐level CMD thresholds to ensure that trial results are appropriately interpreted.

## AUTHOR CONTRIBUTIONS

All authors conceived this orthopaedic commentary touching on current opinion in psychometric methodology; all authors drafted the main manuscript and revised it; all authors edited, read and approved the final manuscript.

## CONFLICT OF INTEREST STATEMENT

The authors declare no conflicts of interest.

## ETHICS STATEMENT

The ethics statement is not available.

## Data Availability

Data Availability Statement is not available.
